# Mechanical characteristics of glioblastoma and peritumoral tumor-free human brain tissue

**DOI:** 10.1007/s00701-024-06009-x

**Published:** 2024-02-23

**Authors:** Jessica Kren, Isabelle Skambath, Patrick Kuppler, Steffen Buschschlüter, Nicolas Detrez, Sazgar Burhan, Robert Huber, Ralf Brinkmann, Matteo Mario Bonsanto

**Affiliations:** 1 Department of Neurosurgery, University Hospital Schleswig-Holstein, Luebeck, Germany; 2 Soering GmbH, Quickborn, Germany; 3 Medizinisches Laserzentrum Lübeck GmbH, Luebeck, Germany; 4 Institute of Biomedical Optics, University of Luebeck, Luebeck, Germany

**Keywords:** Brain tumor, Glioblastoma, Brain tissue, Elasticity, Relaxation, Neurosurgery, Biomechanics

## Abstract

**Background:**

The diagnosis of brain tumor is a serious event for the affected patient. Surgical resection is a crucial part in the treatment of brain tumors. However, the distinction between tumor and brain tissue can be difficult, even for experienced neurosurgeons. This is especially true in the case of gliomas. In this project we examined whether the biomechanical parameters elasticity and stress relaxation behavior are suitable as additional differentiation criteria between tumorous (glioblastoma multiforme; glioblastoma, IDH-wildtype; GBM) and non-tumorous, peritumoral tissue.

**Methods:**

Indentation measurements were used to examine non-tumorous human brain tissue and GBM samples for the biomechanical properties of elasticity and stress-relaxation behavior. The results of these measurements were then used in a classification algorithm (Logistic Regression) to distinguish between tumor and non-tumor.

**Results:**

Differences could be found in elasticity spread and relaxation behavior between tumorous and non-tumorous tissue. Classification was successful with a sensitivity/recall of 83% (sd = 12%) and a precision of 85% (sd = 9%) for detecting tumorous tissue.

**Conclusion:**

The findings imply that the data on mechanical characteristics, with particular attention to stress relaxation behavior, can serve as an extra element in differentiating tumorous brain tissue from non-tumorous brain tissue.

## Introduction

Glioblastoma multiforme (GBM) is one of the most malignant primary tumors in the central nervous system (CNS). The incidence of GBM worldwide varies depending on the report and country, from 0.59 cases per 100,000 person-years to 5 per 100,000 person-years [22, 27, 29, 33]. The prognosis of GBM remains poor, despite medical progress in therapy. As it is a highly invasive and fast-growing tumor of the brain’s glia, the treatment strategy usually includes three pillars, microsurgical resection, radiotherapy, and chemotherapy. In addition, newer procedures, such as tumor treating fields or immunotherapy, may be used. Microsurgical resection, if feasible, is considered a highly important component of therapy.

Quality of life and survival rates depend significantly on resection radicality while sparing healthy and especially eloquent areas [41, 43]. If possible, a gross-total or even supramarginal resection should be aimed to achieve [6, 30]. Meningiomas and brain metastases can generally be distinguished from healthy brain tissue intraoperatively. However, even experienced neurosurgeons can face significant challenges distinguishing gliomas from healthy brain tissue.

For this purpose, the neurosurgical toolbox offers a wide range of technical aids, like neuro-navigation, fluorescent dyes, intraoperative 3D-Ultrasound, Confocal Laser Imaging, and Raman technology [4, 12, 13, 18, 21, 24, 26, 31, 34, 37, 39, 42] In recent years, the surgical landscape in neurosurgery has evolved toward multimodality to balance the error rates of individual systems.

Aside from technical methods, tactile feedback is employed to distinguish between healthy brain tissue and tumorous tissue during the early stages of not only neurosurgical but also tumor resections in general. Experienced neurosurgeons utilize haptic perception, whether consciously or unconsciously, in order to obtain additional information regarding the tissue’s condition. Mechanical properties, such as tissue elasticity significantly contribute to this process.

### Elasticity and stress relaxation behavior of brain tissue

Since there is little data on the intraoperative mechanical characteristics of different brain tumors, this project aimed to investigate the suitability of these very properties for distinguishing between glioblastoma (GBM) and non-tumorous tissue in particular. Biological tissues, including brain tissues, are usually viscoelastic [7–10, 40]. Both elasticity and viscosity are quantitatively measurable variables.

Elasticity describes the time-independent deformability of a tissue when a force is applied to it. Brain tissue is one of the softest tissues in the body, along with fatty tissue. Newer studies and our own research have shown that the elasticity values of brain tissue are in the range of 800—1400 Pa [10]. This means brain tissue is extremely soft and sensitive.

Viscosity describes the damping behavior of a tissue. Because viscosity is difficult to determine in biological tissues due to desiccation, stress-relaxation behavior was determined instead in this work.

In the stress-relaxation experiment, the course of the force or load development for maintaining a deformation is recorded over time. Various studies in recent years have shown that changes in the microenvironment of tumors can lead to a stiffening of the extracellular matrix. Factors such as tenascin-C, overexpression of hyaluronic acid, fibronectin, and brevican play decisive roles here. As a result, tumors usually appear firmer than the surrounding healthy tissue. This also applies to GBMs. [3, 20, 25, 38]

This project aimed to investigate how GBMs differ from healthy brain tissue in terms of elasticity and stress-relaxation behavior, providing the neurosurgeon with additional information about the tissue to be resected in cases of uncertainty.

## Materials and methods

### Population

19 patients with initially diagnosed GBM (n = 10) or recurring GBM (n = 9) were included. All recurring GBM patients received chemo- and radiotherapy. Inclusion criteria were: age over 18 years, capable of consent, tumor in supratentorial location, tumor in non-eloquent area. Exclusion criteria were: serious comorbidities, coagulation disorders or blood thinner intake, and pregnancy.

**Fig. 1:**
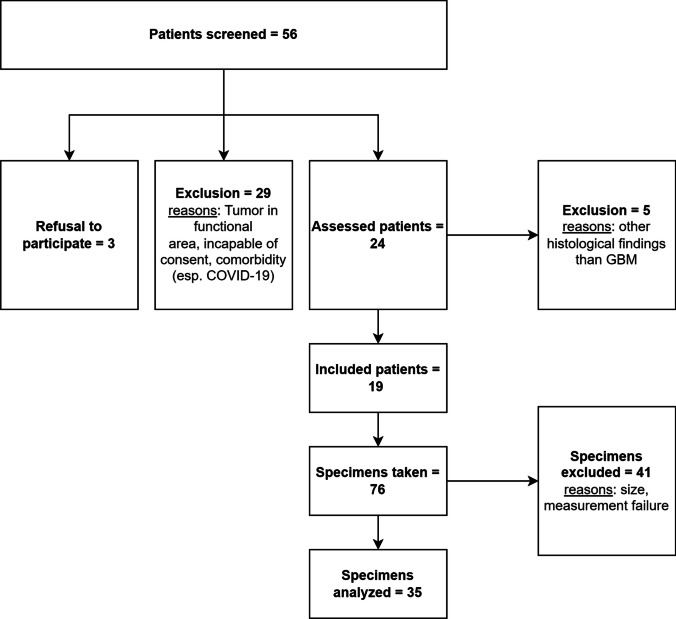
Flow chart of patient recruitment and sample selection

This project was approved by the local ethics committee (Ethics Committee University of Luebeck, AZ 19-319) and carried out according to the Declaration of Helsinki. Patient recruitment and sample selection are illustrated in Fig. 1.

Patient enrolment was carried out as part of regular clinical practice. All patients underwent tumor resection according to current international neurosurgical guidelines and were followed up as part of standard care. In addition, a close-meshed complication screening of the study patients was performed. No complications attributable to study-related procedures were identified. All patients received detailed study information and gave their written informed consent. Detailed patient information is presented in Table 1, 2 and 3.

**Table 1:** Patient's characteristics I

Characteristic	Description	Frequency	Percentage
Sex	Male	11	58%
	Female	8	42%
Recurrence	Yes	9	47%
	No	10	53%
Hemisphere	Left	6	32%
	Right	13	68%
Location	Frontal	8	42%
	Temporal	8	42%
	Parietal	2	10%
	Occipital	1	5%

**Table 2:** Patient's characteristics II

Characteristic		
Age [yrs]	Mean (Median)	58.3 (56.5)
	Range	31 – 80
	Standard Deviation	14.6
Tumor Volume [cm^3^]	Mean (Median)	33.4 (26.8)
	Range	1.0 – 79.7
	Standard Deviation	25.2

**Table 3:** Patient's characteristics in detail

Characteristics in detail					
No.	Sex	Age	Region	Recurrence	Tumor volume [cm^3^]
1	M	58	Frontal	Yes	30.3
2	M	79	Temporal	No	20
3	M	56	Temporal	Yes	23.3
4	M	57	Occipital	Yes	57.1
5	F	80	Temporal	No	9.0
6	M	48	Frontal	Yes	2.9
7	M	59	Frontal	Yes	14.4
8	F	58	Parietal	No	7.8
9	M	34	Frontal	Yes	73.7
10	M	31	Frontal	No	57.6
11	F	77	Temporal	No	1.0
12	M	56	Temporal	Yes	45.7
13	M	73	Frontal	No	60.7
14	F	72	Frontal	No	79.7
15	F	78	Parietal	No	53.1
16	F	32	Frontal	No	5.7
17	M	55	Temporal	Yes	7.9
18	F	60	Temporal	Yes	60.2
19	F	53	Temporal	No	10.5

### Tissue samples

**Fig. 2:**
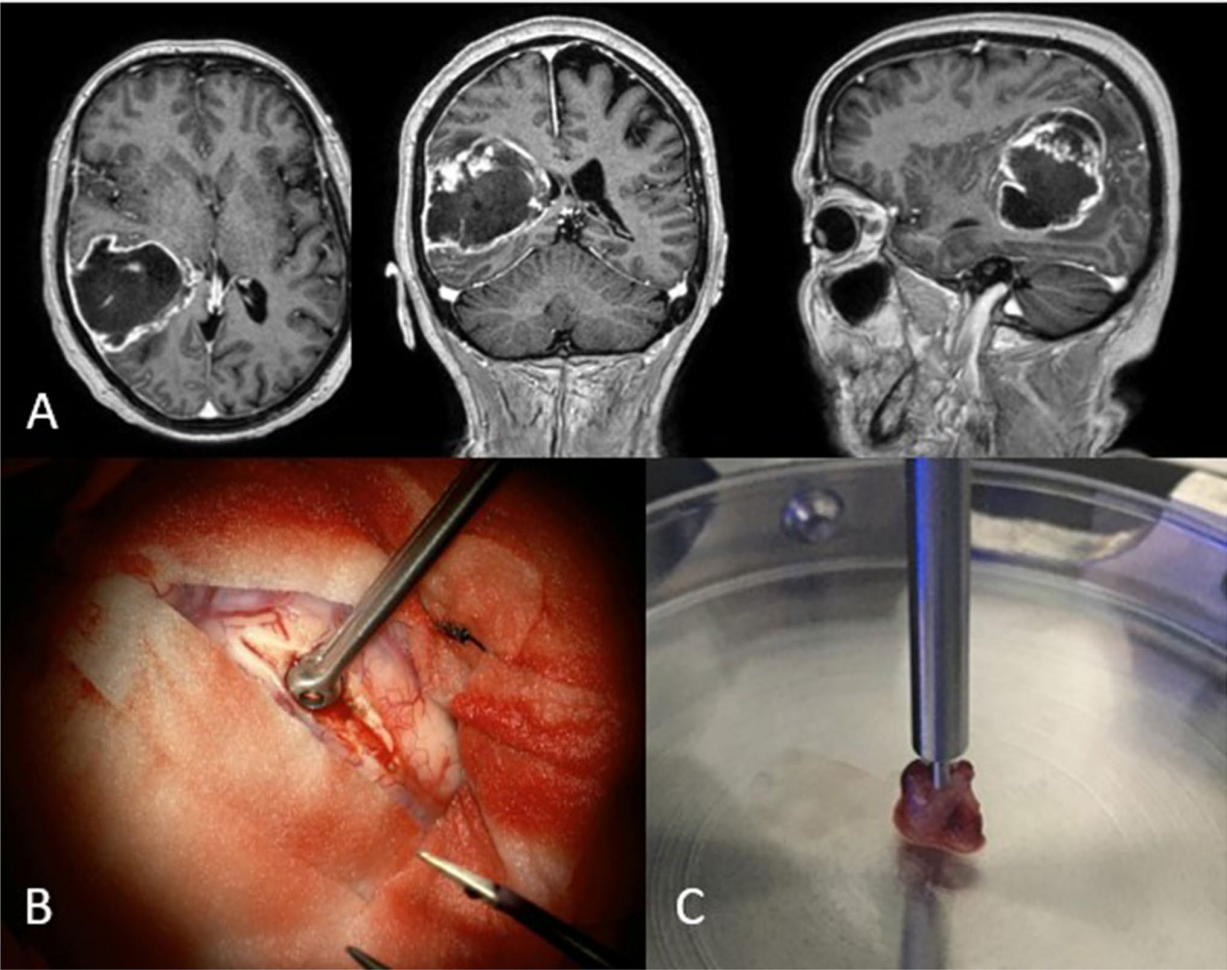
**A** MR-images of a GBM in three axes. **B** Sample collection in the surgical setting using a 5mm tumor grasping forceps. **C** Sample of solid tumor portion placed under the indenter tip

For this project, samples of tumor tissue and, if possible, non-tumorous brain tissue were taken during tumor resections and mechanically measured by indentation. Non-tumorous brain tissue was collected only if it was affected by the resection from the access path or the resection cavity and was not located in eloquent areas. All samples were gathered using a 5 mm grasping forceps (see Fig. 2B). Sampling was initially based on the surgeon's assessment of whether the tissue was tumor or non-tumor.

After sampling, the fresh preparations were measured immediately. Subsequently, all samples were preserved in formalin solution, sectioned, H&E-stained (hematoxylin and eosin) and examined histopathologically by a neuropathologist. The neuropathologist classified all samples into non-tumor (no tumor cells detectable) and tumor (over 60% tumor cells). Samples containing up to 60% tumor cells were excluded. In seven cases, tissue that was considered tumorous by the surgeon was subsequently diagnosed as non-tumorous by the neuropathologist.

### Test setting

**Fig. 3:**
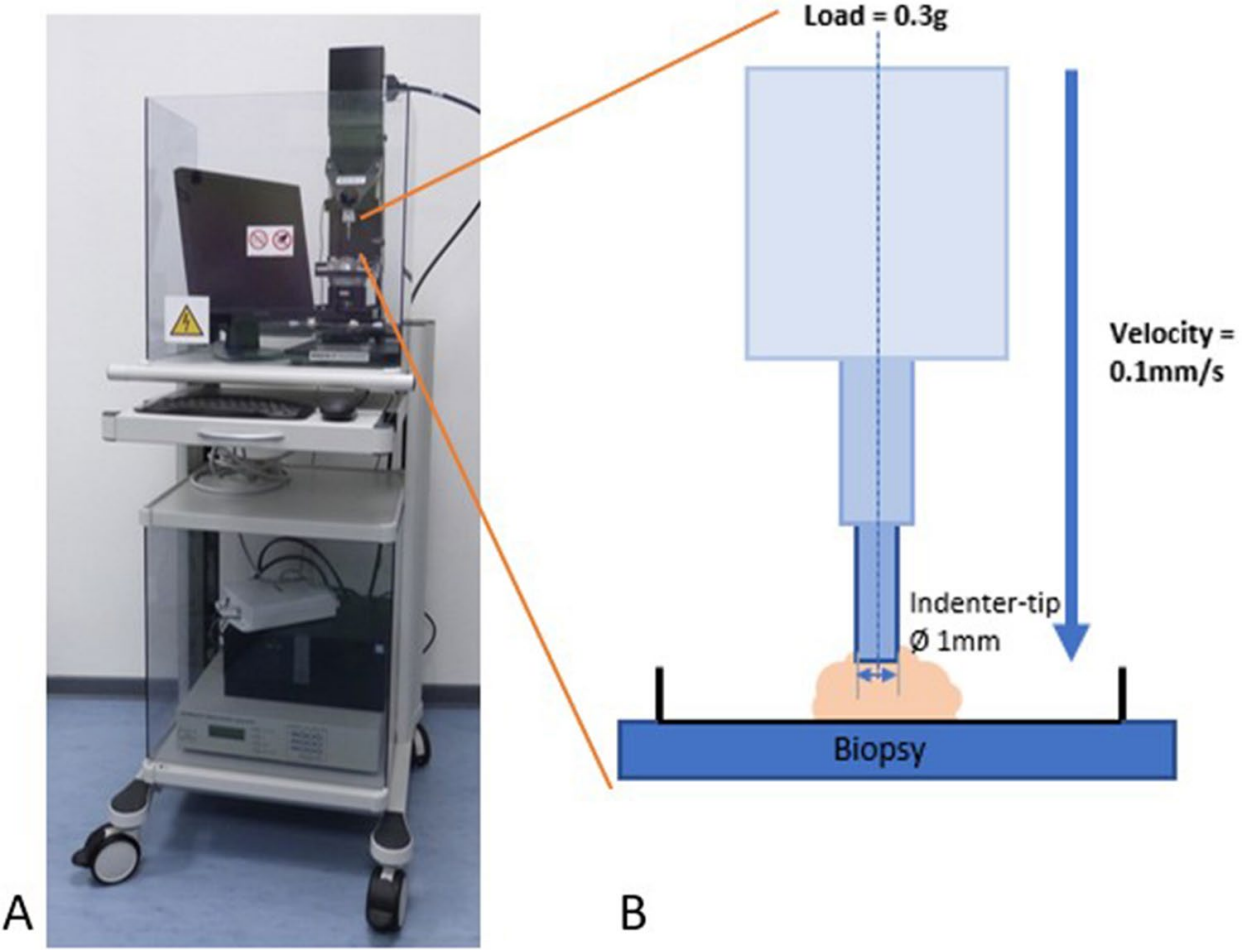
**A** The mechanical tester, which was used to estimate elastic modulus and relaxation behavior. **B** A schematic representation of the measurements

After collection, all samples were immediately measured in the operation room to avoid desiccation effects. The Mach-1 v500c® device from Biomomentum (Montreal, Canada) was used to determine the mechanical properties. The mechanical tester is equipped with a vertical single-axial load cell (up to 0.25N). Using a plane-ended cylindrical test rod (with a diameter of 1 mm), samples were loaded unconfined at a rate of 0.1 mm/s up to a load of 0.3 g (see Fig. 3). Furthermore, the reached position was held for 30 s to determine the stress-relaxation behavior of the tissue.

**Fig. 4:**
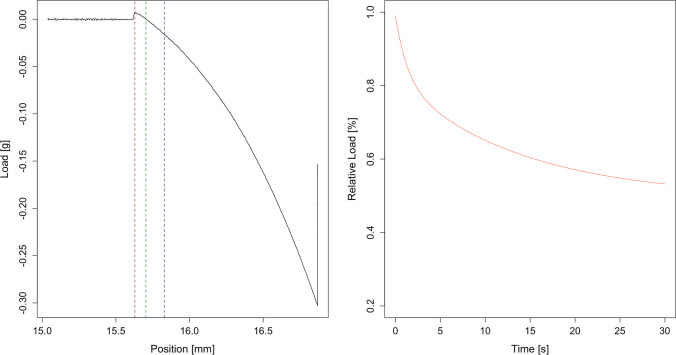
Left: Example of a load-indentation curve. The position represents the vertical position of the indenter. The dashed lines indicate (from left to right): Initial contact to the wet sample surface with an overshoot resulting from a suction effect (red), the estimated tissue contact (green) and at 200μm of indentation. (blue) Right: Example of a relaxation curve

Before every measurement course, a height calibration was performed. Each indentation measurement resulted in a load-indentation diagram (see example in Fig. 4) and a corresponding stress-relaxation curve. The Elastic Modulus was evaluated at 200 μm indentation by determining the slope in the load-indentation diagram.

Each sample was measured three times in different locations. The arithmetic mean value of these measurements was calculated for further analysis. Room temperature was held at 20 °C during the whole experiment. Measurements with a resulting deformation of more than 30% of the initial sample height, as well as measurements where the indenter tip punctured the tissue, were discarded from further analysis to avoid unwanted effects. Elasticity and stress-relaxation behavior of 35 (10 non-tumorous, 25 GBM) samples were examined by indentation.

### Elasticity and relaxation

The following formula was used to calculate the respective Elastic Modulus (Young’s Modulus [E]) proposed by Zhang et al. [44] with the Poisson ratio being *ν* = 0.5. A Poisson ration of *ν* = 0.5 was chosen due to the quasi incompressibility of brain tissue [19] and as a result of our own previous experiments on porcine brain tissue:

1 $$E=P*\frac{(1-{\nu }^{2})}{2*a*w*\kappa (\nu \frac{a}{h})}$$

*P* is the applied force and *w* the indentation depth. The numerical function *2aκ* is a correction term proposed by Hayes et al. [23] with *a* = 1 mm being the indenter diameter. Values for *κ* for plane-ended cylindrical indenters can be taken directly from Hayes et al.

The stress relaxation behavior was observed in a relaxation experiment over 30 s and fitted into the following two-term prony series adapted from Sasaki et al. [36]:

2 $$f\left(x\right)= {G}_{1}*{e}^{(-t*{\tau }_{1})}+ {G}_{2}*{e}^{(-t*{\tau }_{2})}+{G}_{0}$$

### Statistical analysis and data over-sampling

Statistical analysis was conducted using the open-source statistical software R (version 4.3.2 binary for macOS). The Wilcoxon Rank Sum Test was utilized with alpha = 0.05 to examine group differences, while the Spearman Rank Coefficient (r) was applied to determine correlations.

Due to the restrictive requirements for the samples and the limited amount of non-tumorous brain tissue that could be obtained, the current data set is small and somewhat unbalanced. For this reason, the training data was artificially over-sampled and balanced to facilitate the application of machine learning algorithms for classification. The available data set was split randomly into 250 test and training sets (stratified by class). Each test set consists of 4 non-tumorous samples and 10 GBM samples. The data sets for training were subsequently utilized in a logistic regression training process.

Oversampling was performed using a method called’Adaptive Synthetic Sampling Approach for Imbalanced Learning’ in combination with a downstream use of the Tomek-Link algorithm. This process resulted in an individual training set size of 15 non-tumorous and 15 GBM samples each. The over-sampling of the training data itself took place in the cross-validation phase of the machine learning process to avoid positive bias [35]. Principal component analysis was performed to determine which variables from the indentation measurement were useful for classification purposes. Therefore, neither the Elastic Modulus nor the parameter *G*_*0*_ were considered during the classification process. The classification was based on the material-specific constants of each sample (*G*_*1*_*, G*_*2*_*, τ*_*1*_*, τ*_*2*_) extracted from the relaxation behavior fit.

To evaluate model performance, the parameters precision and recall were used. With

3 $$Precision= \frac{True\;Positives}{True\;Positives+False\;Positives}$$

and

4 $$Recall/Sensitivity= \frac{True\;Positives}{True\;Positives+False\;Negatives}$$

## Results

### Elasticity

**Fig. 5:**
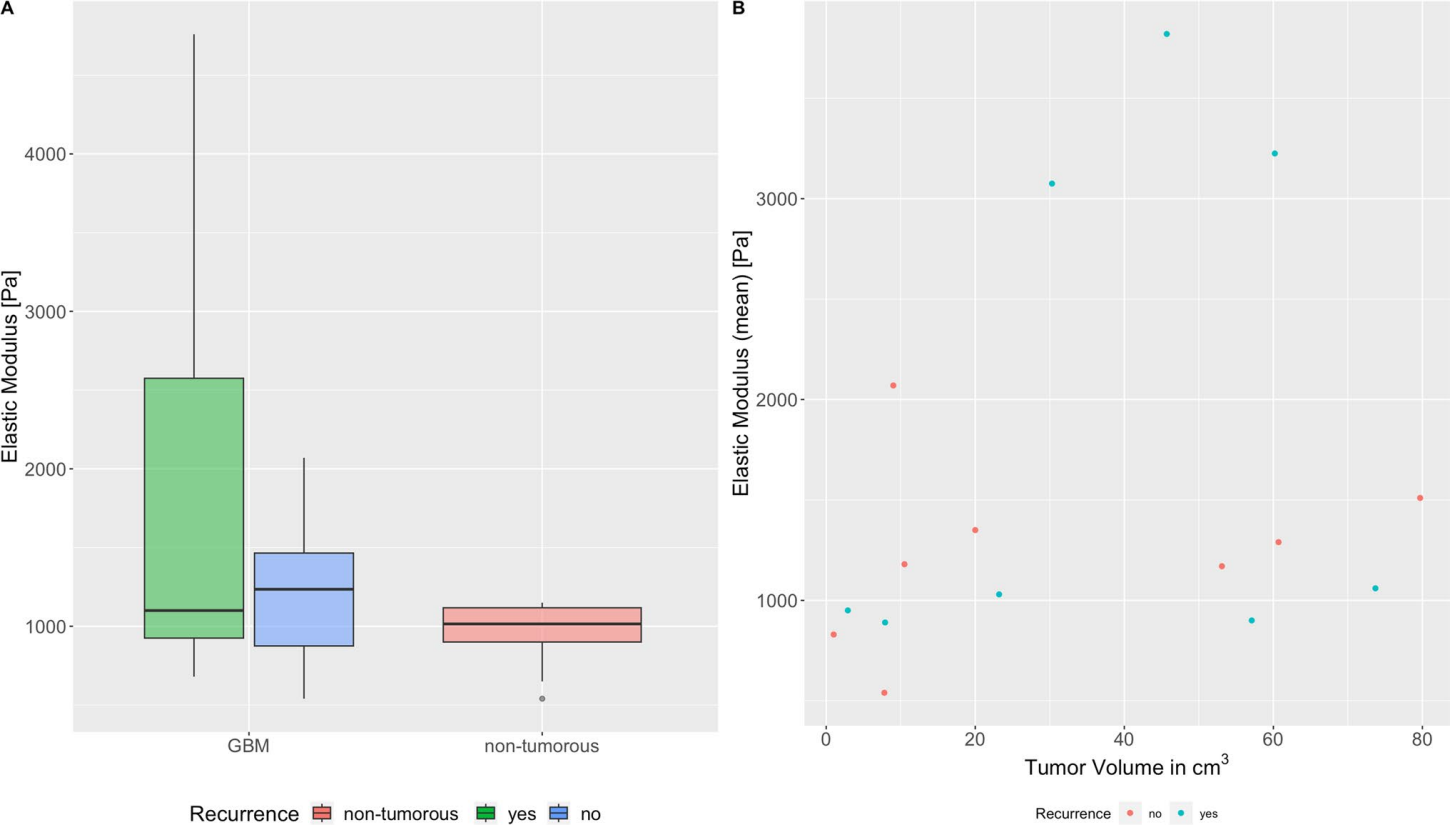
**A** Distribution of Elastic Modulus measurements in recurring (green) and non-recurring GBM (blue) and non-tumorous tissue (red). **B** Elastic Modulus over tumor volume in recurring (blue) and non-recurring GBM (red)

No statistically significant differences were found between the two groups (non-tumor, GBM) with respect to elasticity values. See Table 4 and Fig. 5(A). The GBM group showed a higher spread in Elastic Modulus than the non-tumorous samples and the recurrence group showed a higher spread than the initial diagnosis group (both not significant).

**Table 4:** Results Elasticity

Tissue Type		Elastic Modulus [Pa]
GBM Tissue	Mean (Median)	1541 (1070)
	Range	540 – 4760
	Standard Deviation	1171
Non-Tumorous Brain Tissue	Mean (Median)	956 (1015)
	Range	540 – 1150
	Standard Deviation	210

Additionally, a correlation was found between tumor size and Elastic Modulus (with r = 0.48), which was not statistically significant (p = 0.052). Small tumors tend to be softer than larger ones. See Fig. 5(B).

### Relaxation behavior

**Fig. 6:**
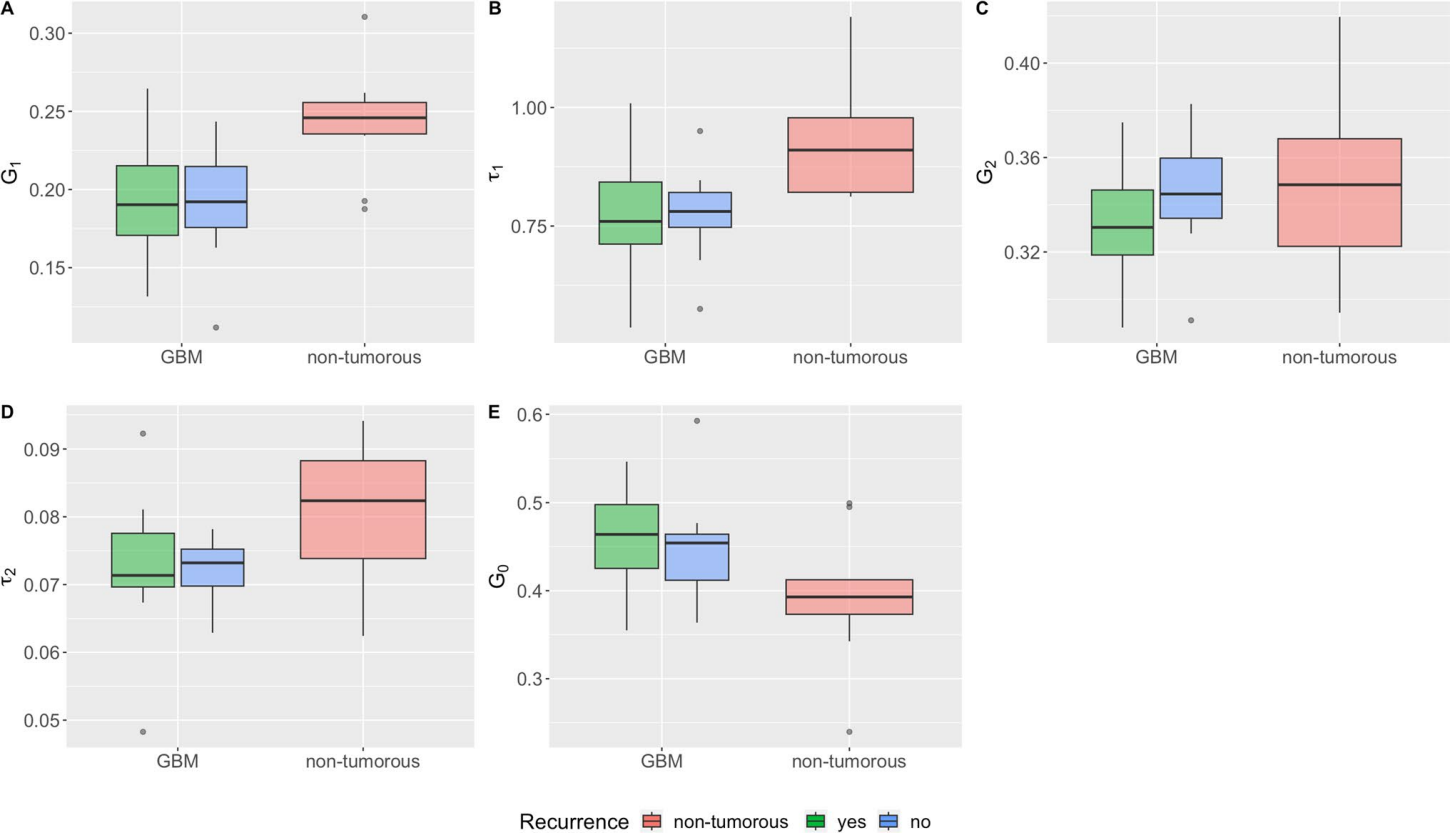
**A**−**E** Distribution of G1, τ1, G1, τ2 and G0 between non-tumorous tissue (red) and GBM tissue (recurring (green) and non-recurring (blue))

The individual relaxation parameters show partially significant group differences. Thus, the differences in *G*_*1*_*, τ*_*1*_*, τ*_*2*_ and *G*_*0*_ between non-tumorous tissue and GBM tissue are significant in the Wilcoxon Rank Sum Test with p = 0.0005 (W = 215) for *G*_*1*_, p = 0.001 (W = 211) for *τ*_*1*_, p = 0.01 (W = 189) for *τ*_*2*_ and p = 0.01 (W = 61) for *G*_*0*_. See Table 5 and Fig. 6.

**Table 5:** Results Relaxation Behavior

Tissue Type	Characteristic		Value
GBM Tissue	*G*_*1*_	Mean (Median) Range SD	0.195 (0.193) 0.112 – 0.265 0.0347
	*τ*_*1*_	Mean (Median) Range SD	0.781 (0.772) 0.536 – 1.01 0.107
	*G*_*2*_	Mean (Median) Range SD	0.339 (0.338) 0.228 – 0.406 0.0277
	*τ*_*2*_	Mean (Median) Range SD	0.0731 (0.0728) 0.0483 – 0.0923 0.00821
	*G*_*0*_	Mean (Median) Range SD	0.451 (0.457) 0.355 – 0.593 0.0578
Non-Tumorous Brain Tissue	*G*_*1*_	Mean (Median) Range SD	0.242 (0.246) 0.188 – 0.310 0.0348
	*τ*_*1*_	Mean (Median) Range SD	0.931 (0.910) 0.812 – 1.19 0.128
	*G*_*2*_	Mean (Median) Range SD	0.348 (0.348) 0.294 – 0.420 0.0394
	*τ*_*2*_	Mean (Median) Range SD	0.0808 (0.0824) 0.0624 – 0.0941 0.0103
	*G*_*0*_	Mean (Median) Range SD	0.394 (0.393) 0.240 – 0.499 0.0739

**Fig. 7:**
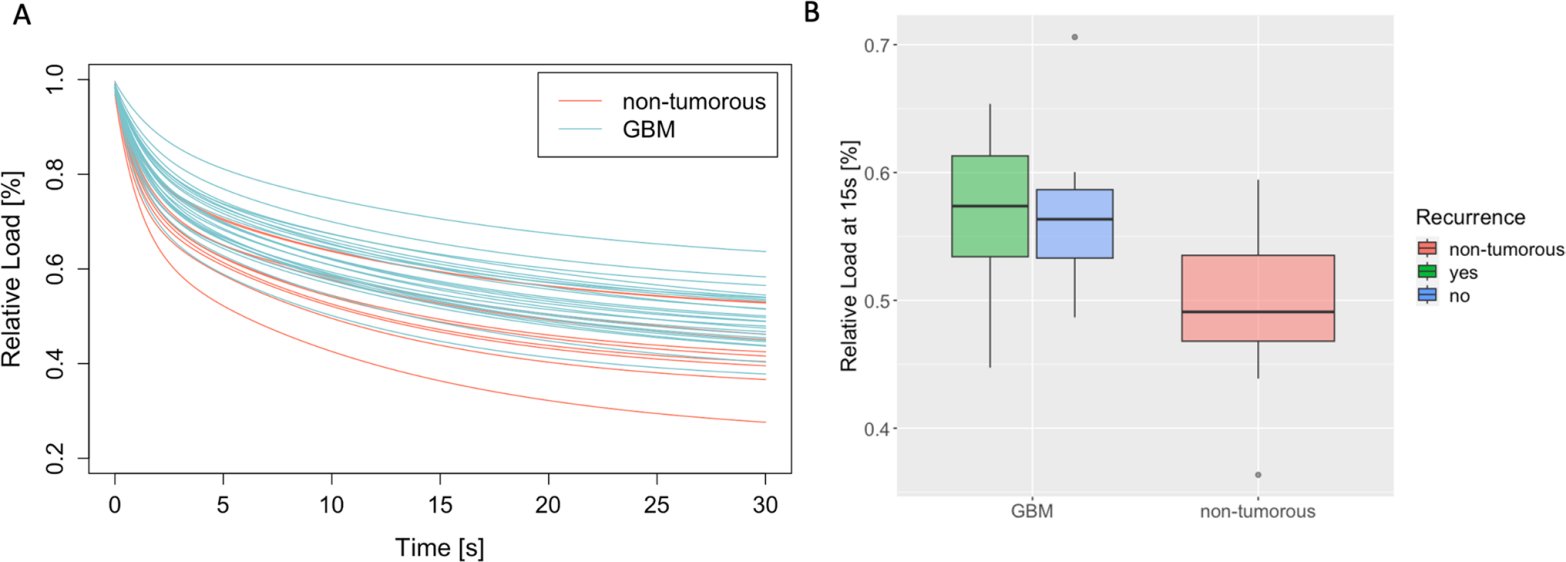
**A** Relaxation behavior measurements in tumorous (blue) and non-tumorous tissue (red). The relaxation behavior experiment was performed over the course of 30s. **B** Relaxation behavior measurements at 15s. Shown is the distribution of relative load in recurring (green) and non-recurring GBM (blue) and non-tumorous tissue (red)

In the stress-relaxation experiment, the course of the force or load development for maintaining a deformation is recorded over time. Non-tumorous tissue appears to relax somewhat faster than GBM tissue. At 15 s, deformation maintenance values are mean = 50% (median = 49%) for non-tumorous tissue and mean = 56% (median = 56%) for GBM tissue of the initial weight load of 0.3 g. See Fig. 7. Group differences in the Wilcoxon Rank Sum Test are significant with p = 0.006 (W = 199). See Fig. 7.

In addition, the height of the samples was examined for group differences (see Table 6). A Wilcoxon Rank Sum Test showed no significant differences in sample height between the two groups.

**Table 6:** Distribution of sample height in non-tumorous and tumorous samples

Tissue type	n	Mean	Median	Range
Non-tumorous	10	3.64	3.54	2.33–5.14
GBM	25	5.15	4.66	1.91–12.87

### Classification

**Fig. 8:**
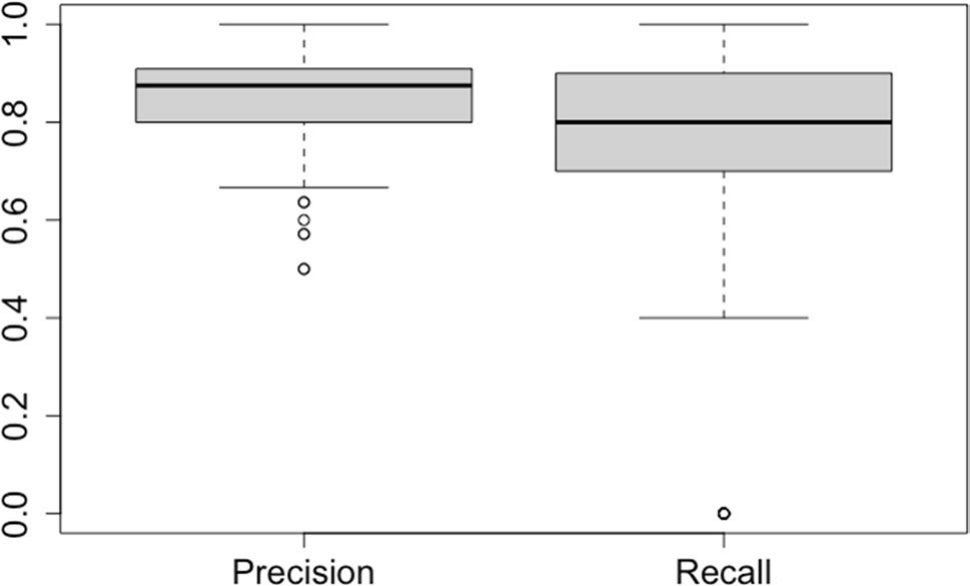
Distribution of Precision and Recall over 250 different test data sets

The aim of classification by logistic regression was to determine, whether a sample is tumorous or non-tumorous tissue, based only on the stress-relaxation behavior of each sample. Recall for the tumorous class was estimated with 0.83 with a standard deviation of SD=0.12 and precision was estimated with 0.87 with a standard deviation of 0.09 (see Fig. 8).

## Discussion

Haptic information serves as a crucial intraoperative information source for surgeons from varied surgical disciplines as it enables them to accurately distinguish between healthy and diseased tissue. Neurosurgeons can easily differentiate tactilely between tumors such as meningiomas, metastases, and other clearly defined brain tumors. However, haptic tactile findings in the border region between glial tumors and healthy brain tissue are more intricate. The interpretation of this sensory stimulus necessitates extensive surgical expertise. Nevertheless, haptics, coupled with other advanced visual measures such as fluorescent dyes, MRI, and intraoperative ultrasound, serve as a crucial augmented source of intraoperative information for the surgeon. The mechanical indentation measurements outlined herein reveal the first direct intraoperative data on elastographic measurements of human CNS tissue, as well as GBM tissue. This study stands in distinction to previously published data on ex vivo indentation measurements of animal CNS samples or post-mortem human CNS tissue [7–10, 17, 40].

Despite the small size of the data set, the results suggest that the information on elasticity and stress relaxation behavior can be used as an additional factor in distinguishing tumorous from non-tumorous tissue. The sensitivity/recall values achieved in the classification (83%) are similar to the known values for 5-ALA (approx. 85%) and exceed those of sodium fluorescein (42–80.8%) [1, 5, 28, 32].

However, although the ranges of elasticity values and stress relaxation behavior partially overlap, trends can be derived that indicate that tumor and peritumoral, non-tumorous tissue differ in their mechanical behavior. Differences between various brain areas in the healthy brain were not considered here, as well as the transition zones from healthy to tumor tissue (which contained less than 60% tumor cells).

The relatively high standard deviations of precision and recall may be due to noise in the measurement and subsequent neuropathological evaluation of the samples. This could be attributed to inconsistencies in the histological analysis of the samples, which did not allow for a 1:1 assignment of the measuring range.

The tissue samples obtained are not regular, healthy brain tissue, but rather from the peritumoral area. However, it is crucial to be able to distinguish this tissue from the tumor tissue during the tumor resection and to spare it. Intraoperative information on the mechanical parameters of the tissue can provide valuable additional information to the surgeon, thus potentially increasing the extent of the resection and prolonging the survival time and the recurrence-free period [6, 30].

In seven cases, tissue that was considered tumorous by the surgeon was subsequently diagnosed as non-tumorous by the neuropathologist. This might be due to inaccuracies of the neuro-navigation and leakage of fluorescein into the peritumoral tissue and also shows the importance of a multimodal intraoperative classification of tumor and non-tumor.

One significant issue with neuro-navigation is its growing inaccuracy during the advancement of surgical interventions and manipulations. Changing of intracranial pressure (brain shift), escaping cerebrospinal fluid (CSF) after dura opening, and manipulations during the resection itself push the accuracy of neuro-navigation to its limits. [37, 39]

To compensate for this problem, the possibility of intraoperative MRI exists. However, this requires high equipment, logistical, and time expenditure. Newer 3D ultrasound systems, developed in recent years, can assist with adapting preoperative MRIs to the surgical site based on the intraoperative situation, by employing anatomical landmarks, such as vessels and other brain structures. The use of ultrasound requires the surgeon to undergo an extended learning process and is relatively time-consuming. [12, 13] One of the latest commercially available techniques for tissue differentiation in neurosurgery is intraoperative confocal laser endoscopy. It also requires a high level of equipment and personnel effort and the use of fluorescein. [4, 34, 42]

Beyond that, Raman technology is currently under investigation on an exclusively experimental scale to determine its potential in differentiating brain tumor tissue from healthy tissue [18, 24, 31]. For improved resection accuracy, additional fluorescent dyes such as fluorescein and 5-aminolevulinic acid can be utilized. These dyes accumulate in tumorous tissue under specific conditions allowing for visualization under special light sources. These dyes are largely ineffective in low-grade gliomas, as they do not typically absorb either contrast medium or the aforementioned dyes [21, 26].

In addition, these substances may not always be free of adverse effects. Uneven aggregation, leakages while excising, and physiological degradation of the dyes over time further complicate intraoperative interpretation.

This study shows similar results to those from previous MRE studies [2]. Despite the similarity of these results, the dependence of the values on the measurement parameters and the preoperative versus intraoperative approach should be emphasized. Other load parameters, such as the load speed and depth, produce different results in viscoelastic tissues, due to their nonlinear behavior, particularly with regard to relaxation behavior.

An interesting future step would be the implementation of an intraoperative method, eliminating the need for biopsies. Successful and precise application could support resection radicality and accuracy. This type of measurement is already available in the form of intraoperative ultrasound elastography. However, the disadvantage is that, as with most ultrasound imaging, a high level of experience is required to interpret the images. Despite promising research findings, the accuracy of this method is directly related to the skills of the surgeon. Moreover, the employment of this technology entails a significant investment in sophisticated equipment and logistical resources. [12, 13] Additionally, the device’s low spatial resolution compromises its precision, rendering it inadequate for microsurgical excision of CNS tumors.

In recent years, Detrez et al. and Burhan et al. have also delivered promising, contactless approaches using optical coherence elastography for tumor delineation in the field of neurosurgery [11, 14–16].

Further research will be needed, especially to consider the variations in mechanical properties of specific brain areas, especially white and grey matter.

## Data Availability

The data can be accessed by contacting the corresponding author.

## References

[1] Acerbi F, Broggi M, Schebesch K, Höhne J, Cavallo C, De Laurentis C, Eoli M, Anghileri E, Servida M, Boffano C, Pollo B, Schiariti M, Visintini S, Montomoli C, Bosio L, La Corte E, Broggi G, Brawanski A, Ferroli P (2018) Fluorescein-guided surgery for resection of high-grade gliomas: a multicentric prospective phase II study (FLUOGLIO). Clin Cancer Res: Off J Am Assoc Cancer Res 24:52–61. 10.1158/1078-0432.CCR-17-1184 10.1158/1078-0432.CCR-17-1184 29018053

[2] Aunan-Diop J, Halle B, Pedersen CB, Jensen U, Munthe S, Harbo F, Andersen MS, Poulsen FR (2022) Magnetic resonance elastography in intracranial neoplasms: a scoping review. Top Magn Reson Imaging 31(1):9–22. 10.1097/RMR.0000000000000292 35225840 10.1097/RMR.0000000000000292

[3] Barnes JM, Przybyla L, Weaver VM, Ewald A (2017) Tissue mechanics regulate brain development, homeostasis and disease. J Cell Sci 130(1):71–82. 10.1242/jcs.191742 28043968 10.1242/jcs.191742 PMC5394781

[4] Belykh E, Miller EJ, Carotenuto A, Patel AA, Cavallo C, Martirosyan NL, Healey DR, Byvaltsev VA, Scheck AC, Lawton MT, Eschbacher JM, Nakaji P, Preul MC (2019) Progress in confocal laser endomicroscopy for neurosurgery and technical nuances for brain tumor imaging with fluorescein. Front Oncol 9:554. 10.3389/fonc.2019.00554 31334106 10.3389/fonc.2019.00554 PMC6616132

[5] Broggi G, Brawanski A, Ferroli P (2018) Fluorescein-guided surgery for resection of high-grade gliomas: a multicentric prospective phase II study (FLUOGLIO). Clin Cancer Res 24(1):52–61. 10.1158/1078-0432.CCR-17-1184 29018053 10.1158/1078-0432.CCR-17-1184

[6] Brown TJ, Brennan MC, Li M, Church EW, Brandmeir NJ, Rakszawski KL, Patel AS, Rizk EB, Suki D, Sawaya R, Glantz M (2016) Association of the extent of resection with survival in glioblastoma: a systematic review and meta-analysis. JAMA Oncol 2(11):1460–1469. 10.1001/jamaoncol.2016.1373 27310651 10.1001/jamaoncol.2016.1373 PMC6438173

[7] Budday S, Nay R, Rooij Rd, Steinmann P, Wyrobek T, Ovaert TC, Kuhl E (2015) Mechanical properties of gray and white matter brain tissue by indentation. J Mech Behav Biomed Mater 46:318–330. 10.1016/j.jmbbm.2015.02.024 25819199 10.1016/j.jmbbm.2015.02.024 PMC4395547

[8] Budday S, Sommer G, Holzapfel GA, Steinmann P, Kuhl E (2017) Viscoelastic parameter identification of human brain tissue. J Mech Behav Biomed Mater 74:463–476. 10.1016/j.jmbbm.2017.07.014 28756040 10.1016/j.jmbbm.2017.07.014

[9] Budday S, Sommer G, Haybaeck J, Steinmann P, Holzapfel GA, Kuhl E (2017) Rheological characterization of human brain tissue. Acta Biomaterialia 60:315–329. 10.1016/j.actbio.2017.06.024 28658600 10.1016/j.actbio.2017.06.024

[10] Budday S, Ovaert TC, Holzapfel GA, Steinmann P, Kuhl E (2020) Fifty shades of brain: a review on the mechanical testing and modeling of brain tissue. Arch Comput Methods Eng 27(4):1187–1230. 10.1007/s11831-019-09352-w

[11] Burhan S, Detrez N, Rewerts K, Strenge P, Buschschlüter S, Kren J, Hagel C, Bonsanto MM, Brinkmann R, Huber R (2024) Phase unwrapping for MHz optical coherence elastography and application to brain tumor tissue. Biomed Opt Express 15:1038–1058. 10.1364/BOE.510020 10.1364/BOE.510020 PMC10890849 38404346

[12] Cepeda S, García-García S, Arrese I, Velasco-Casares M, Sarabia R (2022) Advantages and limitations of intraoperative ultrasound strain elastography applied in brain tumor surgery: a single- center experience. Operative Neurosurgery 22(5):305–314. 10.1227/ons.0000000000000122 35438272 10.1227/ons.0000000000000122

[13] Chan HW, Uff C, Chakraborty A, Dorward N, Bamber JC (2021) Clinical application of shear wave elastography for assisting brain tumor resection. Front Oncol 11:619286. 10.3389/fonc.2021.619286 33732645 10.3389/fonc.2021.619286 PMC7956956

[14] Detrez N, Burhan S, Rewerts K, Kren J, Hagel C, Bonsanto MM, Theisen-Kunde D, Huber R, Brinkmann R (2023) Air-Jet based optical coherence elastography: processing and mechanical interpretation of brain tumor data. In: Optical Elastography and Tissue Biomechanics X Proc. SPIE 12381, Optical Elastography and Tissue Biomechanics X, 1238105. 10.1117/12.2649835

[15] Detrez N, Rewerts K, Matthiae M, Buschschlüter S, Bonsanto MM, Theisen-Kunde D, Brinkmann R (2021) Flow Controlled Air Puff Generator Towards In Situ Brain Tumor Detection Based on MHz Optical Coherence Elastography. Beitrag in European Conference on Biomedical Optics 2021, München, Deutschland

[16] Detrez N, Burhan S, Strenge P, Kren J, Hagel C, Bonsanto MM, Theisen-Kunde D, Huber R, Brinkmann R (2023) Air-jet based optical coherence elastography of brain tumor tissue: stiffness evaluation by structural histological analysis. In Nadkarni SK, Scarcelli G (Hrsg.) Emerging Technologies for Cell and Tissue Characterization II (Band 12629, S. 126290M). SPIE. 10.1117/12.2670944

[17] Dommelen JA, Sande TPJ, Hrapko M, Peters GWM (2010) Mechanical properties of brain tissue by indentation: interregional variation. J Mech Behav Biomed Mater 3(2):158–166. 10.1016/j.jmbbm.2009.09.001 20129415 10.1016/j.jmbbm.2009.09.001

[18] Eichberg DG, Shah AH, Di L, Semonche AM, Jimsheleishvili G, Luther EM, Sarkiss CA, Levi AD, Gultekin SH, Komotar RJ, Ivan ME (2021) Stimulated Raman histology for rapid and accurate intraoperative diagnosis of CNS tumors: prospective blinded study. J Neurosurg 134(1):137–143. 10.3171/2019.9.jns192075 31812144 10.3171/2019.9.JNS192075

[19] Eskandari F, Rahmani Z, Shafieian M (2020) The effect of large deformation on Poisson’s ratio of brain white matter: an experimental study. Proc Inst Mech Eng Part H: J Eng Med 235:401–407 10.1177/0954411920984027 33357009

[20] Fu Z, Zhu G, Luo C, Chen Z, Dou Z, Chen Y, Zhong C, Su S, Liu F (2022) Matricellular protein tenascin C: Implications in glioma progression, gliomagenesis, and treatment. Front Oncol 12:971462. 10.3389/fonc.2022.971462 36033448 10.3389/fonc.2022.971462 PMC9413079

[21] Goryaynov SA, Widhalm G, Goldberg MF, Chelushkin D, Spallone A, Chernyshov KA, Ryzhova M, Pavlova G, Revischin A, Shishkina L, Jukov V, Savelieva T, Victor L, Potapov A (2019) The role of 5-ALA in low-grade gliomas and the influence of antiepileptic drugs on intraoperative fluorescence. Front Oncol 9:423. 10.3389/fonc.2019.00423 31192128 10.3389/fonc.2019.00423 PMC6540822

[22] Gousias K, Markou M, Voulgaris S, Goussia A, Voulgari P, Bai M, Polyzoidis K, Kyritsis A, Alamanos Y (2009) Descriptive epidemiology of cerebral gliomas in northwest Greece and study of potential predisposing factors, 2005–2007. Neuroepidemiology 33(2):89–95. 10.1159/000222090 19494549 10.1159/000222090

[23] Hayes WC, Keer LM, Herrmann G, Mockros LF (1972) A mathematical analysis for indentation tests of articular cartilage. J Biomech 5(5):541–551. 10.1016/0021-9290(72)90010-3 4667277 10.1016/0021-9290(72)90010-3

[24] Ji M, Lewis S, Camelo-Piragua S, Ramkissoon SH, Snuderl M, Venneti S, Fisher-Hubbard A, Garrard M, Fu D, Wang AC, Heth JA, Maher CO, Sanai N, Johnson TD, Freudiger CW, Sagher O, Xie XS, Orringer DA (2015) Detection of human brain tumor infiltration with quantitative stimulated Raman scattering microscopy. Sci Transl Med 7(309):309–163309163. 10.1126/scitranslmed.aab0195 10.1126/scitranslmed.aab0195 PMC4900155 26468325

[25] Khoonkari M, Liang D, Kamperman M, Kruyt FAE, Rijn Pv (2022) Physics of brain cancer: multiscale alterations of glioblastoma cells under extracellular matrix stiffening. Pharmaceutics 14(5):1031. 10.3390/pharmaceutics14051031 35631616 10.3390/pharmaceutics14051031 PMC9145282

[26] Kiesel B, Freund J, Reichert D, Wadiura L, Erkkilae MT, Woehrer A, Hervey-Jumper S, Berger MS, Widhalm G (2021) 5-ALA in suspected low-grade gliomas: current role, limitations, and new approaches. Front Oncol 17(11):699301. 10.3389/fonc.2021.699301 10.3389/fonc.2021.699301 PMC8362830 34395266

[27] Korja M, Raj R, Seppä K, Luostarinen T, Malila N, Seppälä M, Mäenpää H, Pitkäniemi J (2018) Glioblastoma survival is improving despite increasing incidence rates: a nationwide study between 2000 and 2013 in Finland. Neuro-Oncology 21(3):370–379. 10.1093/neuonc/noy164 10.1093/neuonc/noy164 PMC6380416 30312433

[28] Kuppler P, Strenge P, Lange B, Spahr-Hess S, Draxinger W, Hagel C, Theisen-Kunde D, Brinkmann R, Huber R, Tronnier V, Bonsanto MM (2023) The neurosurgical benefit of contactless in vivo optical coherence tomography regarding residual tumor detection: a clinical study. Front Oncol 13:1151149. 10.3389/fonc.2023.1151149 37139150 10.3389/fonc.2023.1151149 PMC10150702

[29] Lee C-H, Jung K-W, Yoo H, Park S, Lee SH (2010) Epidemiology of primary brain and central nervous system tumors in Korea. J Korean Neurosurg Soc 48(2):145–152. 10.3340/jkns.2010.48.2.145 20856664 10.3340/jkns.2010.48.2.145 PMC2941858

[30] Li YM, Suki D, Hess K, Sawaya R (2016) The influence of maximum safe resection of glioblastoma on survival in 1229 patients: Can we do better than gross-total resection? J Neurosurg 124(4):977–988. 10.3171/2015.5.JNS142087 26495941 10.3171/2015.5.JNS142087

[31] Orringer DA, Pandian B, Niknafs YS, Hollon TC, Boyle J, Lewis S, Garrard M, Hervey-Jumper SL, Garton HJL, Maher CO, Heth JA, Sagher O, Wilkinson DA, Snuderl M, Venneti S, Ramkissoon SH, McFadden KA, Fisher-Hubbard A, Lieberman AP, Johnson TD, Xie XS, Trautman JK, Freudiger CW, Camelo-Piragua S (2017) Rapid intraoperative histology of unprocessed surgical specimens via fibre-laser-based stimulated Raman scattering microscopy. Nat Biomed Eng 1(2):0027. 10.1038/s41551-016-0027 28955599 10.1038/s41551-016-0027 PMC5612414

[32] Palmieri G, Cofano F, Salvati LF et al (2021) Fluorescence-guided surgery for high-grade gliomas: state of the art and new perspectives. Technol Cancer Res Treat 20:153303382110216. 10.1177/15330338211021605 10.1177/15330338211021605 PMC8255554 34212784

[33] Philips A, Henshaw DL, Lamburn G, O’Carroll MJ (2018) Brain tumours: rise in glioblastoma multiforme incidence in England 1995–2015 suggests an adverse environmental or lifestyle factor. J Environ Public Health 2018:7910754. 10.1155/2018/7910754 30034480 10.1155/2018/7910754 PMC6035820

[34] Restelli F, Mathis AM, Höhne J, Mazzapicchi E, Acerbi F, Pollo B, Quint K (2022) Confocal laser imaging in neurosurgery: a comprehensive review of sodium fluorescein-based CONVIVO preclinical and clinical applications. Front Oncol 12:998384. 10.3389/fonc.2022.998384 36263218 10.3389/fonc.2022.998384 PMC9574261

[35] Santos MS, Soares JP, Abreu PH, Araujo H, Santos J (2018) Cross-validation for imbalanced datasets: Avoiding overoptimistic and overfitting approaches [research frontier]. IEEE Comput Intell Mag 13(4):59–76. 10.1109/MCI.2018.2866730

[36] Sasaki N (2012) Viscoelasticity - From Theory to Biological Applications. 10.5772/49979

[37] Steinmeier R, Rachinger J, Kaus M, Ganslandt O, Huk W, Fahlbusch R (2002) Factors influencing the application accuracy of neuronavigation systems. Stereotact Funct Neurosurg 75(4):188–202. 10.1159/000048404 10.1159/000048404 11910212

[38] Stewart DC, Rubiano A, Dyson K, Simmons CS (2017) Mechanical characterization of human brain tumors from patients and comparison to potential surgical phantoms. PLoS ONE 12(6):0177561. 10.1371/journal.pone.0177561 10.1371/journal.pone.0177561 PMC5459328 28582392

[39] Stieglitz LH, Fichtner J, Andres R, Schucht P, Krähenbühl A-K, Raabe A, Beck J (2013) The silent loss of neuronavigation accuracy: a systematic retrospective analysis of factors influencing the mismatch of frameless stereotactic systems in cranial neurosurgery. Neurosurgery 72(5):796–807. 10.1227/neu.0b013e318287072d 23334280 10.1227/NEU.0b013e318287072d

[40] Weickenmeier J, Rooij Rd, Budday S, Steinmann P, Ovaert TC, Kuhl E (2016) Brain stiffness increases with myelin content. Acta Biomaterialia 42:265–272. 10.1016/j.actbio.2016.07.040 27475531 10.1016/j.actbio.2016.07.040

[41] Xia L, Fang C, Chen G, Sun C (2018) Relationship between the extent of resection and the survival of patients with low-grade gliomas: a systematic review and meta-analysis. BMC Cancer 18(1):48. 10.1186/s12885-017-3909-x 29306321 10.1186/s12885-017-3909-x PMC5756328

[42] Xu Y, Mathis AM, Pollo B, Schlegel J, Maragkou T, Seidel K, Schucht P, Smith KA, Porter RW, Raabe A, Little AS, Sanai N, Agbanyim DC, Martirosyan NL, Eschbacher JM, Quint K, Preul MC, Hewer E (2023) Intraoperative in vivo confocal laser endomicroscopy imaging at glioma margins: can we detect tumor infiltration? J Neurosurg 1–10. 10.3171/2023.5.jns23546 10.3171/2023.5.JNS23546 37542440

[43] Zeng L, Mei Q, Li H, Ke C, Yu J, Chen J (2021) A survival analysis of surgically treated incidental low-grade glioma patients. Sci Rep 11(1):8522. 10.1038/s41598-021-88023-y 33875775 10.1038/s41598-021-88023-y PMC8055980

[44] Zhang M, Zheng YP, Mak AFT (1997) Estimating the effective Young’s modulus of soft tissues from indentation tests—nonlinear finite element analysis of effects of friction and large deformation. Med Eng Phys 19(6):512–517. 10.1016/s1350-4533(97)00017-9 9394898 10.1016/s1350-4533(97)00017-9

